# Supplementary material for: Darwinian Evolution of Self-Replicating DNA in a Synthetic Protocell
Source: Nat Commun. 2024 Oct 22;15:9091. doi: 10.1038/s41467-024-53226-0 (PMC11494085; doi:10.1038/s41467-024-53226-0)
Supplement: Supplementary file 2 — Description of Additional Supplementary Files [file 41467_2024_53226_MOESM2_ESM.pdf]

Description of Additional Supplementary Files

Supplementary Data 1: Mapping and extraction of the frequency of occurrence of all the point mutations that were detected at a frequency of at least 1% in at least one of the evolutionary rounds.
