# Supplementary material for: Darwinian Evolution of Self-Replicating DNA in a Synthetic Protocell
Source: Nat Commun. 2024 Oct 22;15:9091. doi: 10.1038/s41467-024-53226-0 (PMC11494085; doi:10.1038/s41467-024-53226-0)
Supplement: Supplementary file 4 — Reporting Summary [file 41467_2024_53226_MOESM4_ESM.pdf]

nature portfolio

Corresponding author(s): Christophe Danelon

Last updated by author(s): Sep 13, 2024

Reporting Summary

Nature Portfolio wishes to improve the reproducibility of the work that we publish. This form provides structure for consistency and transparency in reporting. For further information on Nature Portfolio policies, see our Editorial Policies and the Editorial Policy Checklist.

Statistics

For all statistical analyses, confirm that the following items are present in the figure legend, table legend, main text, or Methods section.

n/a	Confirmed	
☐	☒	The exact sample size (*n*) for each experimental group/condition, given as a discrete number and unit of measurement
☒	☐	A statement on whether measurements were taken from distinct samples or whether the same sample was measured repeatedly
☐	☒	The statistical test(s) used AND whether they are one- or two-sided<br>*Only common tests should be described solely by name; describe more complex techniques in the Methods section.*
☒	☐	A description of all covariates tested
☒	☐	A description of any assumptions or corrections, such as tests of normality and adjustment for multiple comparisons
☐	☒	A full description of the statistical parameters including central tendency (e.g. means) or other basic estimates (e.g. regression coefficient) AND variation (e.g. standard deviation) or associated estimates of uncertainty (e.g. confidence intervals)
☐	☒	For null hypothesis testing, the test statistic (e.g. *F*, *t*, *r*) with confidence intervals, effect sizes, degrees of freedom and *P* value noted<br>*Give P values as exact values whenever suitable.*
☒	☐	For Bayesian analysis, information on the choice of priors and Markov chain Monte Carlo settings
☒	☐	For hierarchical and complex designs, identification of the appropriate level for tests and full reporting of outcomes
☒	☐	Estimates of effect sizes (e.g. Cohen's *d*, Pearson's *r*), indicating how they were calculated

Our web collection on statistics for biologists contains articles on many of the points above.

Software and code

Policy information about availability of computer code

Data collection	Data collection for flow cytometry and qPCR was performed using the embedded software of the equipment as indicated in the Methods section.
Data analysis	- Flow cytometry data were analyzed with Cytobank (https://community.cytobank.org/).<br>- For analyzing sequencing data, we used a web-based open-source platform for big data analysis at Usegalaxy.org. Specifically, we mapped paired reads using the BWA software package, merged BAM datasets using the MergeSamFiles tool, to realign indels, we used the BamLeftAlign tool, FreeBayes tool to detect genetic variants, and VCFtoTab-delimited tool for converting VCF to tab-delimited data. Further analysis, such as quantification of mutations above specified thresholds, and corrections of semantical errors on frequency calculations were performed in Excel or in Mathematica (Wolfram Research).<br>- The recorded LC-MS/MS data was analyzed with Skyline.<br>- Fitting kinetics and statistical tests were performed using Excel.<br>- Quantitative PCR data was analyzed using the Quantstudio 5 Software (Thermo Fisher).<br>- Galaxy workflow for mapping and quantifying mutation frequencies is available on GitHub repository: https://github.com/DanelonLab/Illumina-NGS-Mutation-Mapping.

For manuscripts utilizing custom algorithms or software that are central to the research but not yet described in published literature, software must be made available to editors and reviewers. We strongly encourage code deposition in a community repository (e.g. GitHub). See the Nature Portfolio guidelines for submitting code & software for further information.

Data

Policy information about availability of data

All manuscripts must include a data availability statement. This statement should provide the following information, where applicable:

- Accession codes, unique identifiers, or web links for publicly available datasets
- A description of any restrictions on data availability
- For clinical datasets or third party data, please ensure that the statement adheres to our policy

- Data are available in the main manuscript, Supporting Information, Supplementary Data 1.
- Protein mass spectrometry data are available on Panorama Public under ProteomeXchange ID PXD054024 and accession URL: https://panoramaweb.org/qxuWjQ.url.
- Raw NGS sequencing data have been uploaded to ENA (European Nucleotide Archive) under study_ID PRJEB75735.
- Previously published protein structure PDB 2EX3 is available at [https://www.rcsb.org/structure/2EX3].
- Source data are provided with this paper. Raw data in multiple labelled files (Excel and GraphPad) are available within a zipped folder named 'Source Data'.

Research involving human participants, their data, or biological material

Policy information about studies with human participants or human data. See also policy information about sex, gender (identity/presentation), and sexual orientation and race, ethnicity and racism.

Reporting on sex and gender	n/a
Reporting on race, ethnicity, or other socially relevant groupings	n/a
Population characteristics	n/a
Recruitment	n/a
Ethics oversight	n/a

Note that full information on the approval of the study protocol must also be provided in the manuscript.

Field-specific reporting

Please select the one below that is the best fit for your research. If you are not sure, read the appropriate sections before making your selection.

☒ Life sciences ☐ Behavioural & social sciences ☐ Ecological, evolutionary & environmental sciences

For a reference copy of the document with all sections, see nature.com/documents/nr-reporting-summary-flat.pdf

Life sciences study design

All studies must disclose on these points even when the disclosure is negative.

Sample size	No sample-size calculation was performed. Experiments were conducted following the community standards.
Data exclusions	No data were excluded from the analyses.
Replication	Reproducibility of the experimental findings was verified by preparing samples on different days and conducting the same experiments multiple times. Evolution campaign Int-Mut was not repeated. Evolution campaigns Int-WT(1) and Int-WT(2) have been performed by two different persons. Evolution campaign Bulk-WT (10x) was performed once, Bulk-WT (100x) was repeated three times, and Con-WT was repeated twice with two different dilution factors.
Randomization	Not relevant in this study because sample grouping or selection was not required.
Blinding	Blinding was not relevant to our study as no group allocation or randomized trials was required, and possible selection bias is not pertinent in this research.

Reporting for specific materials, systems and methods

We require information from authors about some types of materials, experimental systems and methods used in many studies. Here, indicate whether each material, system or method listed is relevant to your study. If you are not sure if a list item applies to your research, read the appropriate section before selecting a response.

Materials & experimental systems

n/a	Involved in the study
☒	☐ Antibodies
☒	☐ Eukaryotic cell lines
☒	☐ Palaeontology and archaeology
☒	☐ Animals and other organisms
☒	☐ Clinical data
☒	☐ Dual use research of concern
☒	☐ Plants

Methods

n/a	Involved in the study
☒	☐ ChIP-seq
☐	☒ Flow cytometry
☒	☐ MRI-based neuroimaging

Plants

Seed stocks	n/a
Novel plant genotypes	n/a
Authentication	n/a

Flow Cytometry

Plots

Confirm that:

☒ The axis labels state the marker and fluorochrome used (e.g. CD4-FITC).

☒ The axis scales are clearly visible. Include numbers along axes only for bottom left plot of group (a 'group' is an analysis of identical markers).

☒ All plots are contour plots with outliers or pseudocolor plots.

☐ A numerical value for number of cells or percentage (with statistics) is provided.

Methodology

Sample preparation	One microliter of liposome sample was diluted in 149 µL of swelling buffer. The solution was filtered in 5 mL Falcon tubes with cell-strainer caps and pipetted into 96 U-shaped wells for flow cytometry analysis.
Instrument	FACS Celesta flow cytometer (BD Biosciences)
Software	Cytobank (https://community.cytobank.org/)
Cell population abundance	For each sample about 20,000 events were recorded.
Gating strategy	Gating strategy to select liposomes was described in our previous study (ref. 53). We do not provide a figure in the Supplementary Information because we already published the strategy in several papers and the related results are not central to this study.

☐ Tick this box to confirm that a figure exemplifying the gating strategy is provided in the Supplementary Information.
